# Water Consolidation Performance of Acrylic-Polymer-Modified Materials and Their Concrete Impermeability Repair Characteristics

**DOI:** 10.3390/gels9090764

**Published:** 2023-09-19

**Authors:** Dong Yan, Lipeng Lai, Xuedang Xiao, Lei Zhang, Zilong Zhao, Jun Zhao

**Affiliations:** 1 College of Architecture and Civil Engineering, Xinyang Normal University, Xinyang 464000, China; comeonyandong@163.com; 2 Xinyang Lingshi Technology Co., Ltd., Xinyang 464000, China; lingshixxd@163.com; 3 Henan New Environmentally-Friendly Civil Engineering Materials Engineering Research Center, Xinyang Normal University, Xinyang 464000, China; 4 Yellow River Institute of Hydraulic Research, Yellow River Conservancy Commission, Zhengzhou 450003, China; hkyzhanglei@163.com; 5 School of Chemical Engineering and Technology, Sun Yat-Sen University, Zhuhai 519000, China; 6 College of Civil engineering, Zhengzhou University, Zhengzhou, 450001, China; zhaoj@zzu.edu.cn

**Keywords:** grouting material, acrylate, polymer modification, impermeability repair, permeability coefficient

## Abstract

Acrylic materials exhibit favorable grouting repair performance. However, their curing products are easily inclined to drying shrinkage, and their concrete impermeability repair characteristics have seldom been investigated. To improve material properties, reveal the impermeability repair mechanism, and address drying shrinkage, this study proposed the addition of styrene–acrylate copolymer emulsion (styrene–acrylic emulsion) to the grouting material to prepare two-component acrylate grouting materials. Using orthogonal and single-factor tests combined with physical and mechanical properties, the mechanical properties and impermeability repair performance (physical and mechanical properties combined) of grouting materials were analyzed and studied, and the optimal ratio of each component of acrylate grouting materials was determined. Results show that (1) the hydrogel produced by the reaction of sodium methacrylate with hydroxyethyl acrylate has good physical and mechanical properties. (2) With the increase in the accelerator dosage, the setting time of slurry initially decreases and then increases; as the initiator dosage increases, the setting time of slurry decreases, which is negatively correlated with the initiator dosage. (3) Talcum powder can improve the physical and chemical properties of gel and enhance the reliability and durability of acrylate grouting materials, and the comprehensive performance is the best at a dosage of 3%. (4) Styrene–acrylic emulsion can increase the solid content and reduce the volume drying shrinkage when added to grouting materials. The fractured impermeable specimens were repaired by grouting with prepared acrylate grouting materials and cured for 24 h for the impermeability test, and the water pressure for the 24 h impermeability repair was 1.0 MPa. This study’s results provide important reference and basis for revealing the impermeability principle of acrylate grouting materials and evaluating their impermeability.

## 1. Introduction

The impermeability repair of concrete is a common problem in civil and hydraulic engineering. Grouting can enhance the impermeability and integrity of the grouted stratum or building; improve the foundation conditions; and exert impermeable, reinforcing, and water-plugging effects. However, ordinary cement slurry can only plug pores and cracks larger than 0.2 mm in the dam body and dam foundation. Since ordinary cement slurry can only plug large cracks, the problem of microcrack grouting has not been completely solved so far. Chemical grouting can repair fine cracks in concrete. At present, chemical grouting materials mainly include acrylic acid grouting, acrylamide grouting, and epoxy resin grouting, among which acrylic acid grouting materials are the most widely used because of their good effect. Acrylamide grouting materials are mainly used for seepage prevention treatment of fine cracks, but their grout and gel have poor durability and a high price, and a certain toxicity will cause harm to the environment. Epoxy resin grouting material has high strength, shrinkage, and good chemical stability. However, its slurry has high viscosity, low injectivity, and poor adhesion to wet cracks. The above problems reflect the superiority of acrylate grouting materials. Acrylate grouting materials have been widely applied to repair dams, waterproof curtains, weathered strata, and underground works because of their low viscosity, injectability in microcracks (0.02 mm), controllable gelation time, and low permeability coefficient of curing products [1,2,3]. The application potential of acrylate grouting materials presents a gradually increasing trend. Relevant technical studies have achieved considerable progress, and an increasing number of units have participated in various links, such as the research, design, production, and application of acrylate grouting materials [4,5]. The crosslinking agents of most acrylate grouting materials are toxic to some extent, failing to meet environmental protection requirements. Bai et al. [6] synthesized acrylic crosslinking agents through direct esterification to prepare acrylate grouting materials, achieving the safe and environment-friendly production of acrylate grouting materials.

However, acrylate grouting materials have been developed toward a multifunctional direction, accompanied by a good impermeability repair effect. In enhancing the performance of acrylate grouting materials, high costs are required. Magnesium acrylate or calcium acrylate mostly serve as the main agents in the design process, and many design factors related to their waterproofness, impermeability, and physical and mechanical properties are involved. In addition, the technical difficulty is great, posing enormous challenges to research on acrylate grouting materials.

Thus, researchers have conducted considerable research on improving impermeability, physical and mechanical properties, and revealing the impermeability mechanism of grouting materials [7,8]. However, the impermeability of acrylate grouting materials has not been studied in conformity with the actual working conditions, and acrylate grouting materials applied in practical engineering may leak again because of drying shrinkage after repair. Problems needing urgent solutions lie in accurately predicting the impermeability repair characteristics of grouting materials, solving the drying shrinkage of curing products, and understanding the coupling relationship between acrylate grouting materials and foundation repair under the actual working state.

Therefore, by adding low-cost styrene–acrylic emulsion into two-component acrylate grouting materials, an experimental model of impermeability repair with acrylate mortar was established in this study. Then, the impermeability repair performance and physical and mechanical properties of the new grouting material were analyzed and investigated to accurately reveal the impermeability repair characteristics of acrylate grouting materials, solve the problem of secondary seepage caused by the drying shrinkage of curing products, and provide reference for the development and optimization of acrylate grouting materials.

At present, researchers have conducted considerable research on chemical grouting materials. Wagner [9] studied the dynamic rheology of different acrylate grouting materials to determine the setting time and the time to reach maximum strength, but detailed research and analysis on their physical and mechanical properties are still lacking. Abolfazli [10] studied and analyzed the influence of cement grouting and chemical grouting using epoxy resin and polyurethane on the shear behavior of rock joints, but the impermeability characteristics remained unexplored. In revealing the copolymerization and crosslinking behavior of acrylate grouting materials, Qavi [11] used SEM imaging to study the crosslinking and copolymerization of acrylamide at different crosslinking agent ratios and reaction temperatures, which provided a reference for controlling the setting time of grouting materials. Ayman [12] probed into the thermal stability of materials through hot pressing, which could be referenced to study the durability of grouting materials. Amal and Bhadani [13,14] explored the swelling property of acrylate hydrogel with synthetic solvent, providing a reference for controlling gel swelling. Using acrylamide and zirconium acetate as the gel system, Zhao G [15] studied and analyzed the cross-linking reaction of acrylamide. John [16] investigated the permeability characteristics of polymer slurry in sand, which laid a foundation for studying the grouting stability of polymer slurry. Alina [17] analyzed the crosslinking mechanism of acrylic gel using dynamic rheology and FTIR technology, which provided a reference for exploring the influence of catalysts on setting time.

Jiang [18] determined the influence of inorganic fillers on acrylate waterproof materials and studied the corrosion, tensile strength, tear strength, and the elongation at break of waterproof materials, which provided a reference for the alkali resistance of acrylate. Liao [19] studied the elongation of acrylate grouting materials and the bonding properties of concrete, which provided a reference for studying the interfacial bonding properties of acrylate. Li and Wang [20,21] studied and analyzed the influence of the water content of slurry on curing products based on the water swelling and water-loss-induced drying shrinkage characteristics of the curing products of acrylate grouting materials, which provided a reference for guiding the application potential of acrylate grouting materials. Based on the characteristics of acrylate spraying film materials, Pan [22] modified acrylate spraying film by polyvinyl alcohol solution, which provided a reference for the self-repair of acrylate spraying film. In improving the water absorption and swelling ratio of acrylate hydrogel, Xu [23] proposed a double-main-agent acrylate hydrogel, which could be referenced to improve the swelling properties of grouting materials. Gu [24] prepared a composite waterproof material with magnesium acrylate/calcium acrylate, providing a reference for improving the water-swelling ratio of gel and the compressive strength of consolidated sand. Jiang [5] modified acrylate grouting materials using epoxy resin, which could be referenced to improve the consolidated sand strength of grouting materials.

The abovementioned research results are primarily aimed at the multifunctionality and physical and mechanical properties of acrylate grouting materials, but their impermeability characteristics have been less investigated, not to mention the research work on the repair of concrete cracks through acrylate grouting. In this study, mortar test blocks were repaired through grouting with acrylate grouting materials, based on which a test model of acrylate grouting repair was established. On the basis of the grouting repair characteristics of grouting materials, the impermeability repair, permeability coefficient, consolidated sand strength, and water-swelling ratio of the developed double-component acrylate grouting materials were, respectively, discussed, and the impermeability coupling relations of acrylate grouting materials in concrete cracks were deduced, providing a basis for optimizing and testing grouting materials.

The remainder of this paper is organized as follows: In Section III, the physical and mechanical properties are investigated through the three-factor four-level orthogonal test scheme, and the optimal proportion of acrylate grouting materials is determined. In Section IV, the effects of accelerator and initiator on the setting time of grouting materials and the effect of inorganic fillers on the alkali resistance of grouting materials are, respectively, studied; the dosage of accelerator, initiator, and inorganic fillers contributing to the optimal performance of grouting materials is obtained; and impermeable mortar specimens are fabricated to conduct the impermeability repair test. In the final section, the whole paper is summarized, and relevant research conclusions are presented.

## 2. Result Analysis and Discussion

### 2.1. Analysis of Orthogonal Test Results

On the basis of the test scheme shown in Section 2.1, the orthogonal test results were acquired, which are shown in Table 1.

The range analysis of the orthogonal test results (Table 2, Table 3 and Table 4) shows that the factors affecting the permeability coefficient of grouting materials were ranked as B (percentage of crosslinking agent in component A) > C (percentage of acrylate in component A) > A (percentage of styrene–acrylic emulsion in component B); the factors affecting the consolidated sand strength of grouting materials were ranked as B (percentage of crosslinking agent in component A) > C (percentage of acrylate in component A) > A (percentage of styrene–acrylic emulsion in component B); and the factors affecting the water-swelling ratio of grouting materials were ranked as B (percentage of crosslinking agent in component A) > C (percentage of acrylate in component A) > A (percentage of styrene-acrylic emulsion in component B). By analyzing Table 2, Table 3 and Table 4, the crosslinking agent had the greatest influence on the mechanical properties of grouting materials. Considering that the crosslinking agent served as a bridge among acrylate molecules, polyacrylate molecules were bonded and crosslinked, which changed the internal structure of the curing product, made the spatial gel network in the curing product of acrylate compact, and restricted the action of water molecules, thereby improving the permeability of acrylate grouting materials. When the dosage of crosslinking agent increased, the number of crosslinking points in the gel increased, the network space was reduced, and the physical and chemical interaction between networks was enhanced, which enhanced the compressive strength of consolidated sand and reduced the water-swelling ratio. When the concentration of acrylate solution increased, the electrostatic repulsion between ions increased, which reduced the water retaining capacity of the gel, thereby decreasing the water-swelling ratio.

Based on the analysis results shown in Table 2, Table 3 and Table 4, the proportion of crosslinking agent in component A had an evident influence on the mechanical properties of grouting materials. Studies have shown that the permeability of grouting materials and consolidated sand strength are influenced greatly by the use of crosslinking agents. As shown in Table 3, the permeability coefficient decreased gradually with the increase in crosslinking agent dosage. Considering the increase in the dosage of crosslinking agent, the cross-linking points in the gel gradually increased, and the intermolecular pore size was smaller. Consequently, water molecules could not flow freely into the gel, and they were even absorbed by the gel. The crosslinking density of the gel was the main factor affecting the consolidated sand strength. With the increase in the dosage of crosslinking agent, the crosslinking density of the gel increased, and the physical or chemical interaction among networks was enhanced, thereby improving the compressive strength of consolidated sand. However, the excessive addition of crosslinking agent would decrease the water-swelling ratio of the gel, which further resulted in poor impermeability.

The compressive strength of solid sand can be improved by increasing the amount of crosslinking agent in component A. When the proportion of the main agent used in component A of the grout is determined, increasing the amount of crosslinking agent can improve the compressive strength of the solid sand body of grout. This is because the crosslinking agent acts as a bridge between the acrylate molecules and makes the polyacrylate molecules cross-link with each other. When the amount of crosslinking agent increases, the number of crosslinking points in the gel increases, the network space decreases, and the physical and chemical interaction between the networks is enhanced, which makes the compressive strength of the solid sand body increase. However, the crosslinking agent should not be used too much. With the increase in crosslinking agent, the space of acrylate solidified substance becomes dense, the action of water molecules is limited, and the expansion rate of solidified substance in water is reduced, thus affecting the impermeability of slurry repair.

Based on the analysis shown in Table 2 and Table 3, the styrene–acrylic emulsion exerted smaller effects on the permeability coefficient of grouting materials and the consolidated sand strength than the main agent and crosslinking agent. Styrene–acrylic emulsion, as a type of water-soluble polymer emulsion, has good compatibility and durability with concrete, and this emulsion is often used to prepare waterproof coatings. When added to grouting materials, styrene–acrylic emulsion can enhance the durability of grouting materials and increase the solid content of the gel, contributing to a low shrinkage ratio and reducing the risk of repeated leakage. When the content of styrene–acrylic emulsion was 35% lower than that in component B, the solid content of the gel was low, while the shrinkage ratio was high, accompanied by the risk of secondary leakage after drying. In the case of excessively high viscosity of the styrene–acrylic emulsion, the viscosity of grouting materials was affected, thereby influencing the grouting effect of the slurry.

Considering the comprehensive properties of acrylate grouting materials, the 15th group in the orthogonal test exhibited excellent physical and mechanical properties and impermeability.

### 2.2. Effect of the Redox System on the Properties of Acrylate Grouting Materials

**Figure 1:**
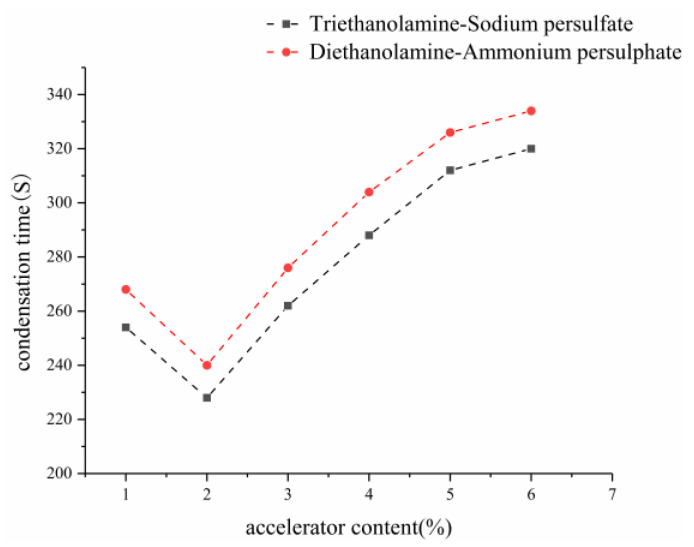
Effect of accelerator on the setting time.

**Figure 2:**
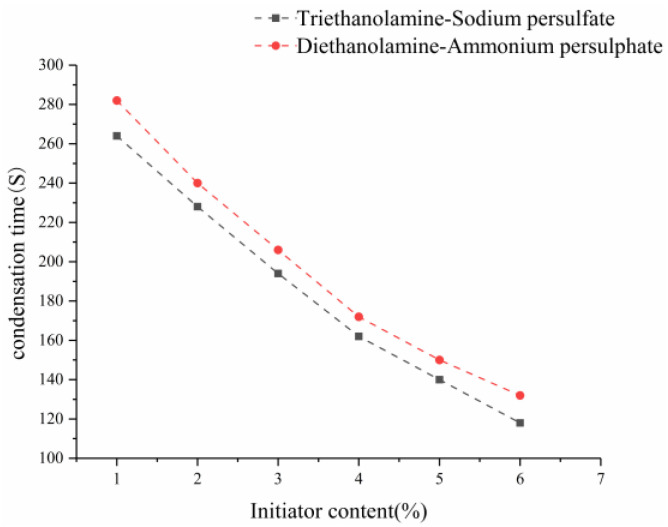
Effect of initiator on the setting time.

Based on the test method shown in Section 2.1, the 15th group with good physical and mechanical properties and impermeability was selected from the orthogonal test, and the effects of different redox systems, such as triethanolamine–sodium persulfate and diethanolamine–ammonium persulfate, on the setting time and water-swelling ratio were investigated. Figure 1 and Figure 2 show the influence of accelerator and initiator on the setting time, respectively.

The variation characteristics of setting time of two groups of different redox systems with the content of accelerator and initiator were plotted through experiments. As shown in Figure 1, the setting time initially decreased and then increased with the increase in the content of accelerator. The accelerator has a dual effect on the decomposition of free radicals by initiator; that is, it can not only guide the initiator to generate free radicals but also neutralize them to reduce free radicals and prolong curing time. With less accelerator content, its neutralization with the initiator decreased. In this case, the concentration of free radicals was high, the polymerization rate was fast, and the curing time was short. When the content of accelerator was less than 2%, its promoting effect on the decomposition of free radicals by initiator decreased, the concentration of free radicals decreased, the polymerization rate slowed down, and the curing time was prolonged.

Figure 2 shows the influence of initiator on the setting time. At a fixed content of accelerator, the curing time decreased with the increase in the content of initiator, because the higher the initiator content, the more free radicals are produced, the faster the polymerization rate, and the shorter the curing time. At a fixed content of accelerator, the setting time decreased with the increase in the content of initiator. As shown in Figure 1 and Figure 2, the setting time of the triethanolamine–sodium persulfate redox system was shorter than that of the diethanolamine–ammonium persulfate at the same contents of accelerator and initiator. The oxidation of sodium persulfate is stronger than that of ammonium persulfate, and the initiation efficiency of triethanolamine–sodium persulfate is higher, which results in the easy decomposition of free radicals, thereby accelerating the curing reaction rate and reducing the setting time of the slurry. The setting time of the slurry can be controlled by the amount of accelerators and initiators in the slurry. The accelerators and accelerators in the grout will affect the setting time of the grout. With the increase in the accelerant content, the setting time of the grout will first decrease and then increase. Because the accelerator has a double effect on the decomposition of free radicals by the initiator, it can not only guide the initiator to generate free radicals but also neutralize the reaction with free radicals so that the free radicals are reduced and the curing time is prolonged. When there is less accelerator, the neutralization between it and the initiator is reduced, the concentration of free radicals is larger, the polymerization rate is faster, and the curing time is reduced. The larger the initiator content, the more free radicals produced in the slurry, the faster the polymerization rate, and the shorter the curing time. However, the accelerator should not be used too much, and when the accelerator is used too much, the volume of the solidified body is obviously expanded after water, the strength is reduced, and damage occurs. Through comprehensive consideration, diethanolamine accounting for 2% of component A was taken as accelerator, and ammonium persulfate accounting for 2% of component B was taken as initiator. Moreover, the setting time of the slurry was 240 s.

The change in the effective component ratio of acrylate grouting materials will affect the physical and mechanical properties of acrylate curing products. By using 1%, 2%, 3%, 4%, and 5% of accelerator and reductant, the influence of the water-swelling ratio of the gel on the 15th group was investigated. Table 5 and Table 6 display the influence of accelerator and initiator on the water-swelling ratio, respectively.

**Figure 3:**
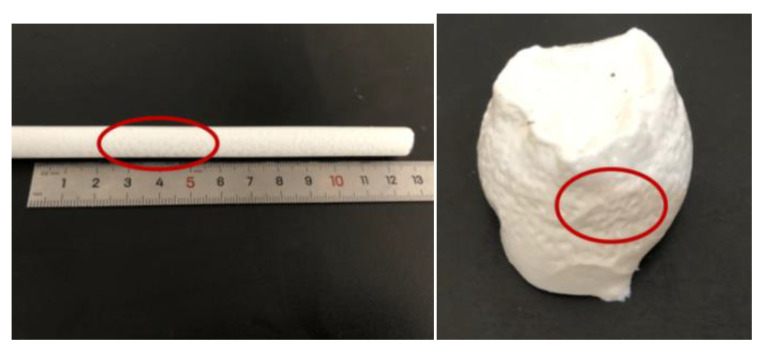
Pores appearing in gel.

Twelve groups of specimens were soaked in distilled water for 7 days, and they kept absorbing water and swelling until reaching an equilibrium. As shown in Table 6 and Table 7, the higher the content of accelerator and oxidant, the greater the water-swelling ratio of the gel. However, the setting time of the gel was lengthened with the increase in the content of accelerator. When the content of initiator increased, the gel volume expanded, and the strength was enhanced. In addition, the initiator was damaged after the water molecules were absorbed to a certain extent. The excessively high content of initiator also shortened the setting time, and the slurry contained a lot of pores after setting (Figure 3), thereby affecting its physical and mechanical properties.

### 2.3. Effect of Inorganic Fillers on the Properties of Acrylate Grouting Materials

**Figure 4:**
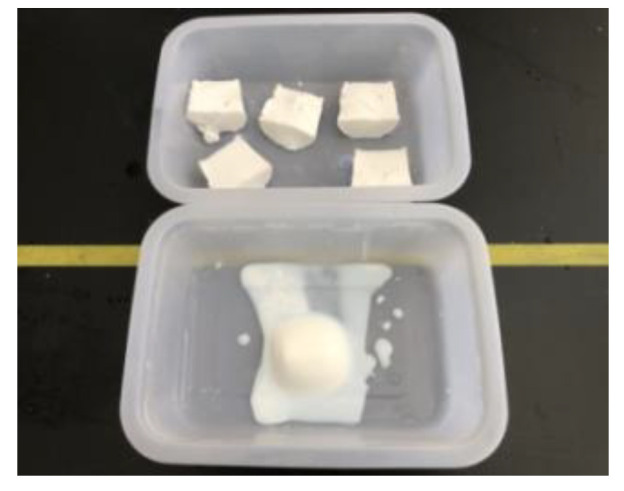
Effect of silica fume on alkali resistance.

**Figure 5:**
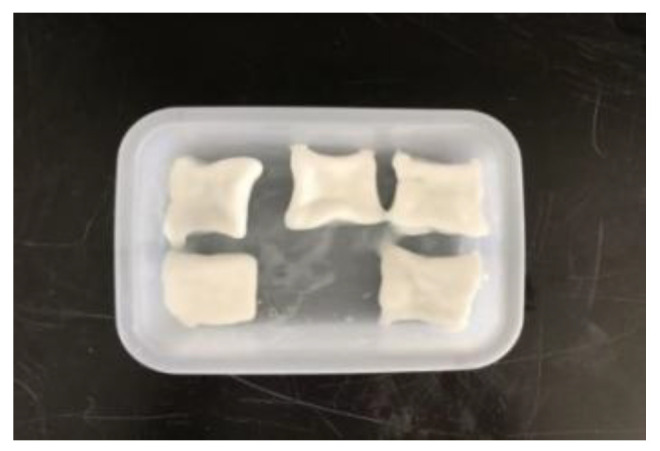
Effect of fumed silica on alkali resistance.

**Figure 6:**
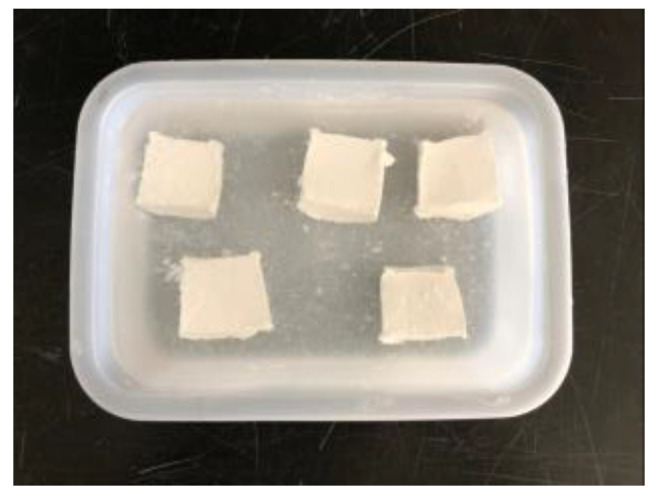
Effect of talcum powder on alkali resistance.

Fillers can improve the physical and chemical properties of gel and improve the reliability and durability of acrylate grouting materials. In studying the influence of fillers on acrylate grouting materials, silica fume, fumed silica, and talcum powder were selected to investigate the influence of 1%, 2%, 3%, 4%, and 5% content on the alkali resistance of grouting materials. Figure 4 shows the effect of silica fume on the alkali resistance of grouting materials; Figure 5 displays the effect of fumed silica on the alkali resistance of grouting materials; and Figure 6 presents the effect of talcum powder on alkali resistance.

The figures show that talc powder performed better than fumed silica and silica fume in affecting the alkali resistance of acrylate grouting materials. Given the active chemical properties, silica fume and fumed silica reacted with accelerator and initiator in the slurry, reducing the effective components in the slurry and resulting in the degradation of its alkali resistance. Talcum powder exhibited stable chemical properties and good toughness. As the content of talcum powder increased, acrylate curing products showed stronger corrosion resistance against the alkaline solution (pH = 13).

Silica fume performed poorly in alkali resistance. After soaking in an alkaline solution for 24 h, the gel was torn and dissolved, resulting in serious corrosion, which became increasingly serious with the increase in the content of silica fume. At a silica fume content of 5%, the gel was seriously corroded, and the specimen was damaged and lost its performance. The alkali resistance of fumed silica was average, and different degrees of corrosion were observed when the filler content was 1–5%. When the content was 1%, the edge of the specimen was torn and corroded, and the volume increased. At a content of 5%, the alkali resistance was improved, but corrosion still occurred at the edge, and the volume did not change significantly. Fumed silica had a small particle size, so its specific surface area was large, and its viscosity was high after adding the slurry, which affected the permeability of the slurry. The alkali resistance of talcum powder was the best. With the increase in filler content, the alkali resistance was gradually improved. When the content was 3% or higher, the gel was in good condition without evident corrosion.

Considering the good performance of talcum powder in the alkali resistance of the slurry, the effects of talcum powder content as a single-factor variable on the viscosity, water-swelling ratio, and consolidated sand strength of grouting materials were investigated.

**Figure 7:**
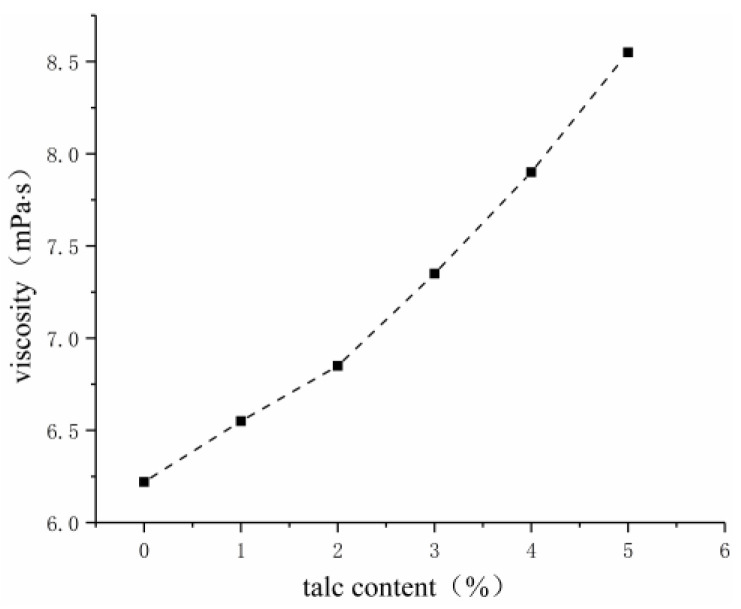
Effect of talcum powder on viscosity.

**Figure 8:**
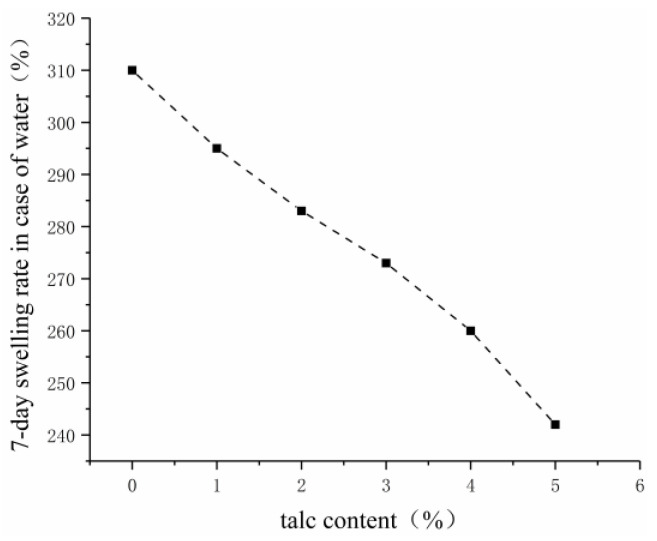
Effect of talcum powder on the water-swelling ratio.

**Figure 9:**
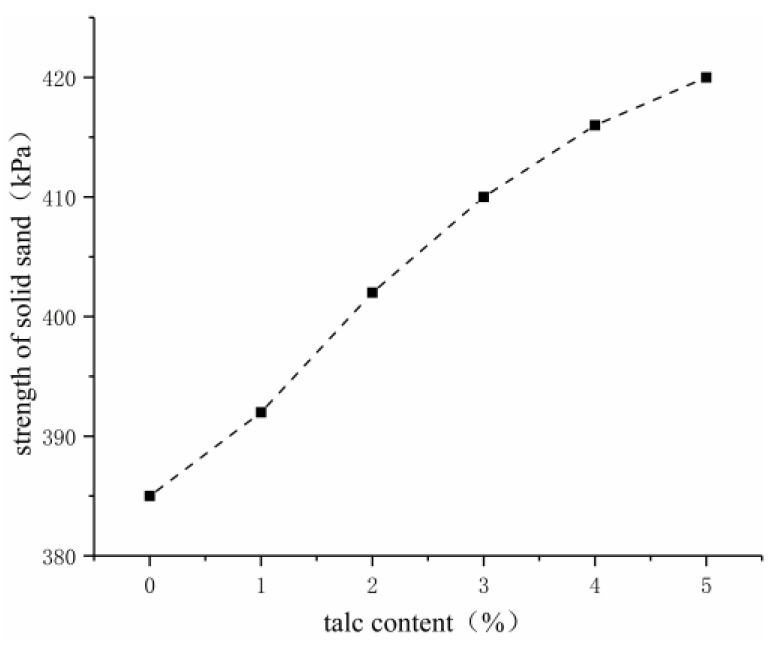
Effect of talcum powder on consolidated sand strength.

As shown in Figure 7, Figure 8 and Figure 9, the water-swelling ratio of the gel decreased after talcum powder was added into the grouting material, and it gradually decreased with the increase in talcum powder content. Given the addition of talcum powder, the gel material became compact, and talcum powder occupied the partial space between the crosslinking points of acrylate molecules, which reduced the water molecules contained in the hydrogel and its water-swelling ratio. The higher the content of talcum powder, the more compact the gel and the greater the consolidated sand strength. Talcum powder, which has good suspension and dispersion properties, can play a skeleton role as a filler to improve strength and stability, as well as increase adhesion. As an inorganic filler, talcum powder can improve the physical and mechanical properties of acrylate grouting materials and enhance the alkali resistance of consolidated sand, but excessive use will increase the viscosity of the slurry, reduce its permeability, and affect the grouting effect. Three percent was selected as the content of talcum powder by fully considering its alkali resistance, viscosity, water-swelling ratio, and consolidated sand strength.

### 2.4. Water Seepage Repair Test of Acrylate Grouting Materials

**Figure 10:**
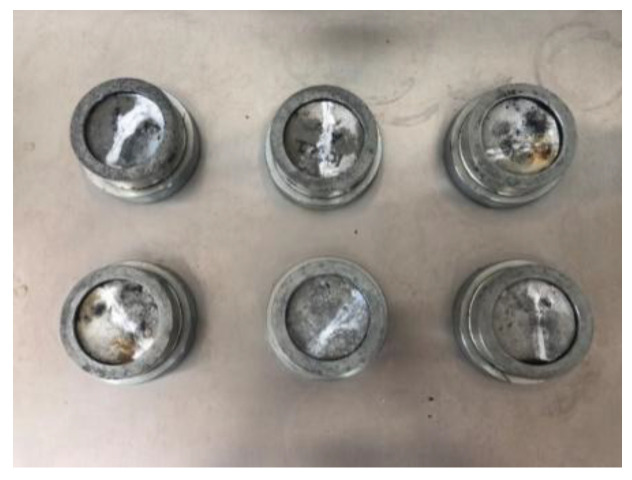
Impermeability repair test of the mortar test block.

**Figure 11:**
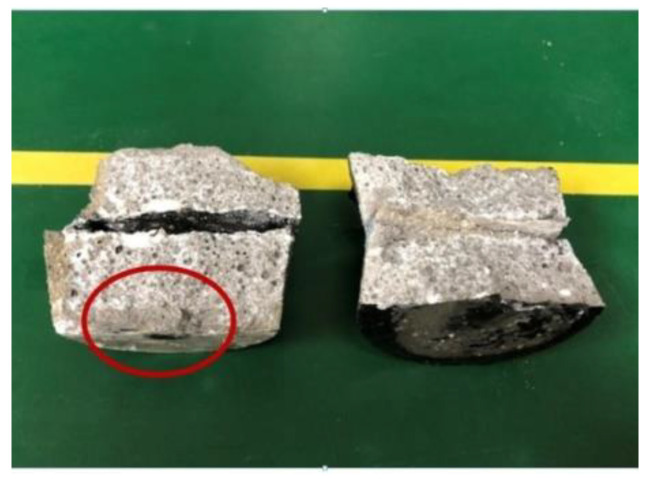
Grouting repair of the mortar test block.

**Figure 12:**
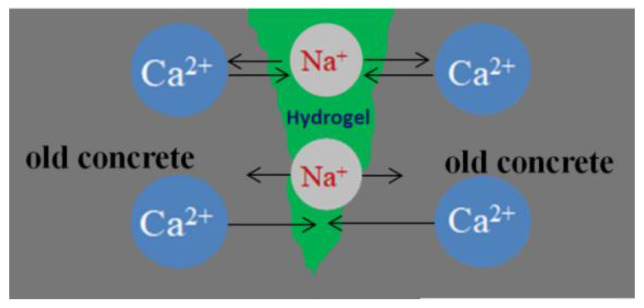
Schematic diagram of chemical complexation.

The impermeability repair performance of the 15th formula was experimentally investigated. The experimental situation showed that the mortar test block after grouting repair was subjected to water seepage along the crack under the action of water pressure (Figure 10), and the leaking mortar test block was broken to observe the grouting repair situation (Figure 11). As shown in Figure 11, under pressure, water flew from the bottom of the specimen through the gel to the top to form a leakage point. The grouting effect of the slurry along the cracks of the specimen was good, and the cracks were completely filled. After being broken, the specimen fractured inside the gel, and the bonding strength between the surface gel and the mortar specimen was higher than that of the material itself. Chemical adsorption and physical adsorption occurred between the cured acrylate and concrete base surface. Chemical adsorption is attributed to high-strength chemical bonding triggered by the chemical complexation and formation of chemical bonds (Figure 12) under complexation reaction between Na^+^ in curing products and Ca^2+^ in mortar specimens. With regard to physical adsorption, the slurry enters the capillary pores in the specimen along cracks because of the capillary action, intermolecular action, and hydrogen bonding. After solidification, the slurry is embedded into concrete like an anchor, which enlarges the contact area between curing products and the mortar specimen, generating physical bonding and considerably enhancing the bonding force between curing products and concrete.

In a dry environment, the water molecules between the acrylate molecules will decrease due to evaporation, resulting in volume shrinkage of the cured material. The drying shrinkage of acrylate solidified material may lead to releakage. The addition of styrene–acrylic emulsion to the slurry can increase the solid content of the slurry, reduce the volume shrinkage of the consolidated body, and ensure the stability of the slurry grouting repair. This is because the styrene–acrylic emulsion as a water-soluble polymer emulsion has good compatibility and durability with concrete, adding it to the grouting material can improve its durability and increase the gel solid content. With free water, the styrene–acrylic emulsion enters between the cross-linking points of the consolidated body, reduces the interpenetrating pores in the consolidated body, increases the solid content, reduces the volume shrinkage, and improves the repair effect of grouting materials on the gap. However, the viscosity of styrene–acrylate emulsion is too high, and excessive addition will affect the viscosity of grout material, thus affecting the grouting effect of its grout.

**Figure 13:**
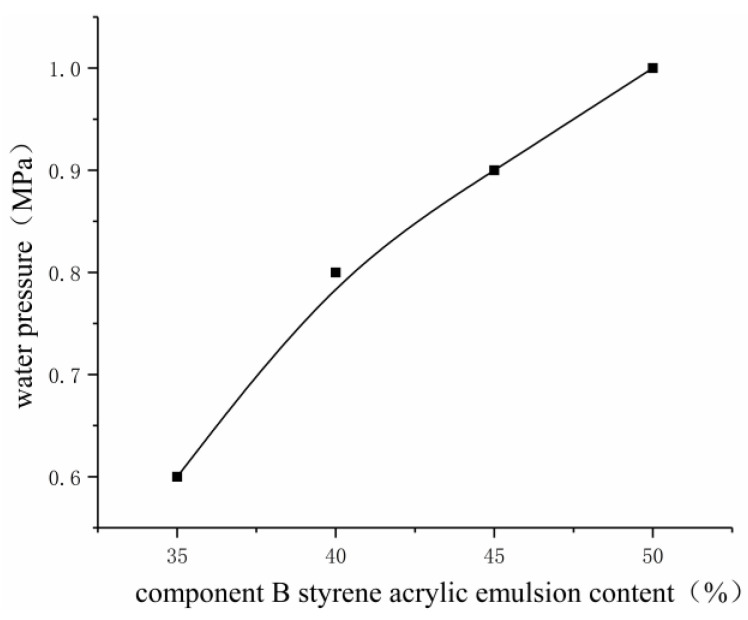
Effect of styrene–acrylic emulsion content on impermeability repair.

Based on the impermeability test of the styrene–acrylic emulsion at different contents (Figure 13), the seepage pressure of grouting repair showed an overall rising trend with the increase in styrene–acrylic emulsion content. At a styrene–acrylic emulsion content of 50%, the impermeability repair effect was enhanced sharply, and the seepage pressure was 1.0 MPa. After the impermeability test on mortar specimens for 3, 7, and 14 days, the acrylate curing product was bonded with concrete cracks to form a whole, and the curing product lost water, while the impermeability was not degraded. As the content of styrene–acrylic emulsion increases, it enters between the crosslinking points of the gel with free water, reducing the pores in the gel, increasing the solid content, mitigating volume shrinkage, and enhancing the plugging and repair effect of grouting materials on cracks. After being added, the styrene–acrylic emulsion increased the solid content of the gel and reduced its volume shrinkage. Through drying–wetting cycles, the curing product did not leak again because of volume shrinkage.

The water plugging and impermeability mechanism of acrylate grouting materials can be divided into filling, bonding, and swelling. Acrylate grouting materials, which are characterized by low viscosity and strong permeability, can be used for filling repair along concrete cracks and cutting off the seepage channel. The chemical adsorption and physical adsorption between the acrylate gel and concrete can contribute to the tight connection between the gel and concrete, and the gel material can be embedded into concrete cracks for bonding the crack interface. The acrylate grouting material will swell in water, and the volume swelling ratio can reach as high as 300%, completely filling the voids between cracks to block the seepage channel.

## 3. Conclusions

In improving the properties of acrylate grouting materials, solving the drying shrinkage of curing product, and revealing the impermeability repair characteristics of acrylate grouting materials starting from acrylate, crosslinking agent, and styrene–acrylic emulsion, orthogonal and single-factor tests were combined in this study to explore the effects of the mix proportion of double-component acrylate grouting materials and the content of talcum powder and styrene–acrylic emulsion on the properties of grouting materials and analyze their impermeability repair characteristics. Finally, the following conclusions were drawn:

(1) When sodium methacrylate accounted for 20% and crosslinking agent for 25% in component A and styrene–acrylic emulsion accounted for 50% in component B, the permeability coefficient of the prepared two-component acrylate grouting material was 2.007 × 10^−8^ cm/s; the consolidated sand strength was 385 kPa; and the water-swelling ratio in 7 days was 320%, all of which indicated good physical and chemical properties.
(2) At a fixed content of initiator, the setting time initially decreased and then increased with the increase in accelerator content, and the setting time was the shortest when the accelerator content was 2% in component A. At a fixed content of accelerator, the setting time decreased with the increase in initiator content, and the setting time was 118 s when the initiator content was 6% in component B. Based on the engineering characteristics, the setting time of the slurry can be reasonably adjusted by adjusting the content of accelerator and initiator.
(3) As the content of talcum powder increased, the solidified grouting material showed remarkable alkali resistance. Meanwhile, with the increase in talcum powder content, the compressive strength of consolidated sand was improved, reaching the highest value of 420 kPa, and the water-swelling ratio was reduced, reaching the lowest value of 242%. The increase in talcum powder content led to the increase in slurry viscosity, thereby affecting its permeability. Through comprehensive consideration, the content of talcum powder was determined to be 3%.
(4) In the impermeability repair test, the two-component acrylate grouting material was characterized as follows: The repair impermeability test specimen started water seepage along cracks under the action of water pressure, and water drops were gradually formed at a low speed as pressurization was continued. Then, the 24 h grouting impermeability repair test was performed when the percentages of sodium methacrylate, hydroxyethyl acrylate, and diethanolamine in component A were 20%, 25%, and 2%, respectively, and the percentages of styrene–acrylic emulsion and ammonium persulfate in component B were 50% and 2%, respectively. The water pressure for impermeability repair was measured as 1.0 MPa. After 3, 7, and 14 days of impermeability tests, the grouting material did not experience performance degradation because of the drying shrinkage of curing products.

In this study, the indoor test and theoretical study were combined, and the impermeability repair test method of adding styrene–acrylic emulsion as a water-soluble polymer emulsion into acrylate grouting materials and grouting repair mortar specimens was proposed. The impermeability test of grouting materials was simplified and closer to the actual situation on site, which had certain references for the subsequent research and development of acrylate grouting materials. The field test and construction technology were not considered because of the lack of actual use data at the project site. Therefore, in future research, a practical engineering test will be conducted on the developed two-component acrylate grouting material, and the construction technology will be optimized to improve the comprehensive performance of acrylate grouting materials. s. In future tests, fly ash, MgO, and PVA fibers will be used as admixtures to investigate the their effects on the wear resistance, crack resistance, pore structure, and fractal characteristics of hydraulic concrete.

## 4. Materials and Methods

### 4.1. Test Scheme

For the orthogonal test scheme, the initial proportion of the slurry was determined by three-factor and four-level orthogonal tests (Table 7). Taking the mass ratio of acrylate and crosslinking agent in component A and that of styrene–acrylic emulsion in component B as the three factors of the orthogonal test, the permeability coefficient, sand consolidation strength, and water-swelling ratio of each group at each proportion were investigated. The percentage of accelerator in component A and that of initiator and retarder in component B were fixed at 1%, 2%, and 0.02%, respectively. In this research stage, the mass ratio of components A and B was 1:1.

For the test scheme of the setting time, the 15th formula with a good performance in the orthogonal test was selected, and the effects of the initiator on the setting time and water-swelling ratio of grouting materials were studied at an accelerator percentage of 1% and initiator percentages of 1%, 2%, 3%, 4%, and 5%. In addition, the effects of the accelerator on the setting time and water-swelling ratio of grouting materials were explored at an initiator percentage of 1% and accelerator percentages of 1%, 2%, 3%, 4%, and 5%.

For the modification test scheme of inorganic fillers, the 15th formula was used, and the effects of setting time on the alkali resistance, viscosity, and consolidated sand strength of grouting materials were studied by taking the mass ratio of 1250-mesh talcum powder in the grouting material as 1%, 2%, 3%, 4%, and 5%.

For the test scheme of grouting repair, the 15th formula with a good performance in the orthogonal test adopted was used, the fractured mortar specimens were subjected to grouting repair, and mortar impermeability tests were performed after curing to explore the influence of the content of styrene–acrylic emulsion on the impermeability repair effect.

### 4.2. Test Materials and Instruments

In this study, sodium methacrylate, hydroxyethyl acrylate, and triethanolamine were selected as component A, and the styrene–acrylate copolymer emulsion, deionized water, ammonium persulfate, and potassium ferricyanide were selected as component B. Silica fume, fumed silica, and talcum powder were used as inorganic fillers.

The main instruments used in this study included a variable-head permeameter, electrohydraulic pressure tester, agitator, impermeability tester, rotary viscosimeter, and thermostatic drying oven.

### 4.3. Permeability Coefficient Test Method

**Figure 14:**
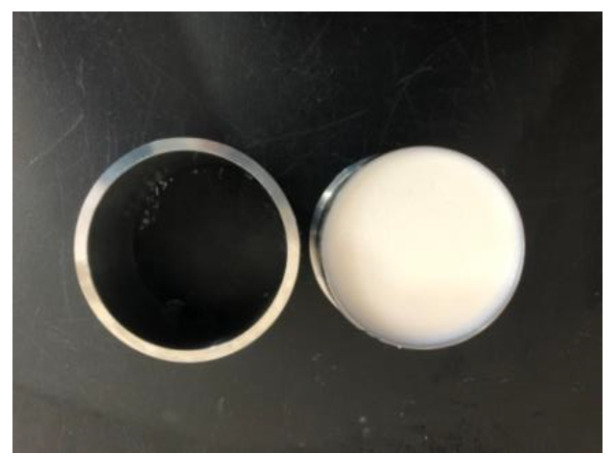
Permeation cutting ring.

**Figure 15:**
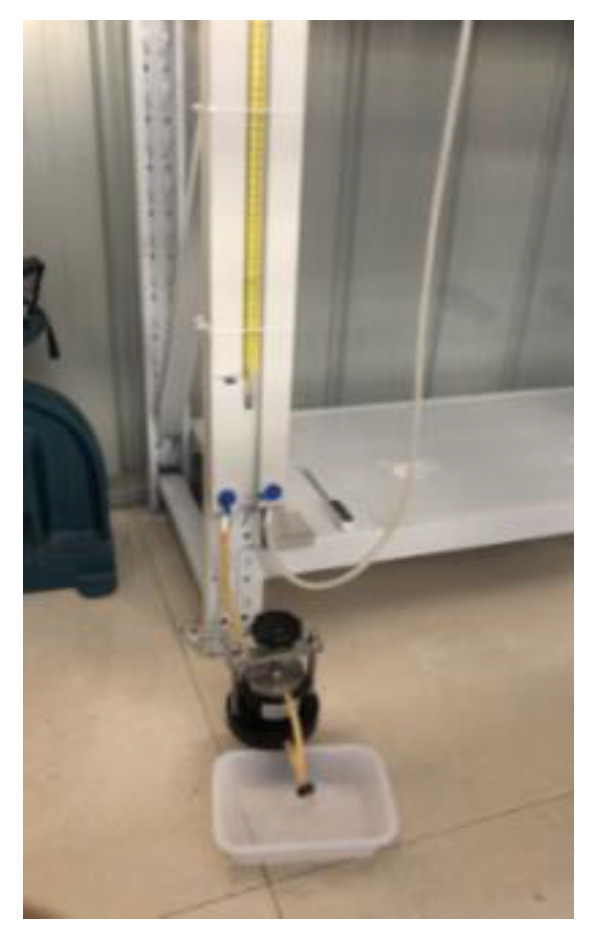
Variable-head permeameter.

The permeability coefficient test was performed in accordance with the Standard for Geotechnical Testing Method. The prepared acrylate slurry was poured into a cutting ring (Figure 14) and formed curing products in the cutting ring. After standing for 1 day, the cutting ring filled with curing products was installed in a permeator, followed by the permeability test through the variable head permeability test method (Figure 15). The permeability coefficient was calculated as follows:

$$k_{T}=2.3\frac{aL}{A\left(t_{2}-t_{1}\right)}lg\frac{H_{1}}{H_{2}}$$

where *k_T_* is the permeability coefficient of the specimen in cm/s; *a* is the cross-sectional area of the variable head pipe in cm^2^; *L* is the permeation diameter in cm; *H*_1_ is the starting head in cm; *H*_2_ is the ending head in cm; *t*_1_ and *t*_2_ are the starting and ending time of head reading, respectively, in s; and 2.3 is the conversion coefficient of *ln* and *lg*.

### 4.4. Test Method for the Compressive Strength of Consolidated Sand

**Figure 16:**
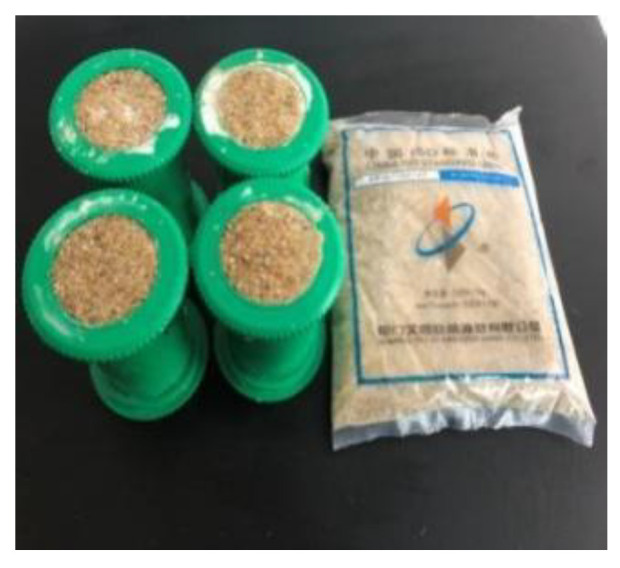
Compressive strength test.

**Figure 17:**
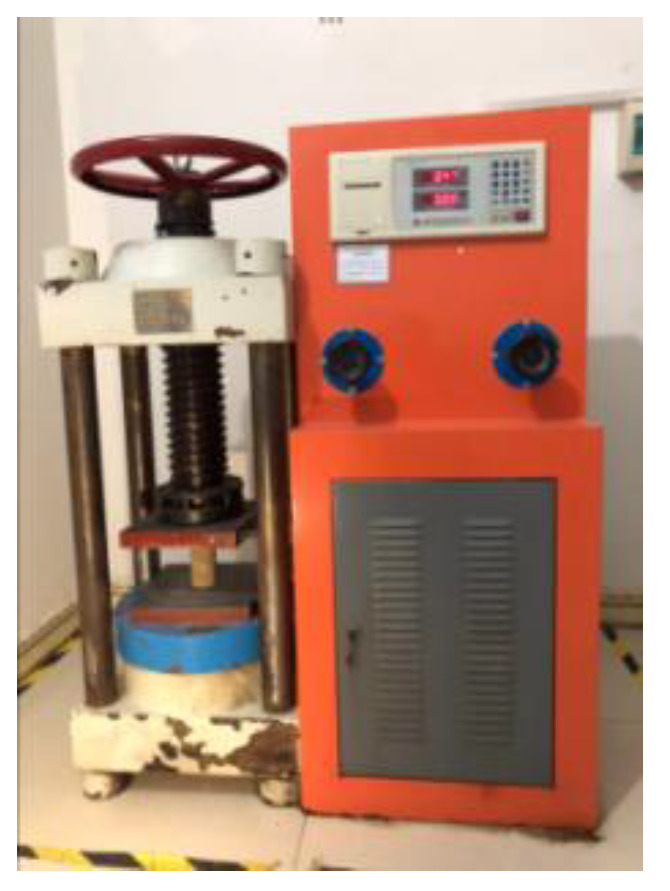
Electrohydraulic pressure testing machine.

A test mold with an inner diameter of 40 mm and a height of 100 mm was used to form a cylindrical consolidated sand body with a diameter of 40 mm and a height of 100 mm. Six specimens were included in each group of the compressive strength test of consolidate sand. The evenly mixed standard sand was initially placed into the test mold, with its surface slightly exceeding the top surface of the test mold, and then the top of the prepared mortar was slowly poured into sand, which was stopped when slurry overflowed the top (Figure 16). After slurry gelation, the surface was floated along the edge of the test mold, and the surface was covered by a preservative film for curing. After 24 h, the test mold was removed, and the compressive strength was determined (Figure 17). The compressive strength of consolidated sand was calculated as follows:

$$\sigma =\frac{P}{A}\times 1000$$

where σ is the compressive strength in kPa; *P* is the failure load in N; and *A* is the pressure-bearing area in mm^2^.

### 4.5. Test Method for Water-Swelling Ratio

**Figure 18:**
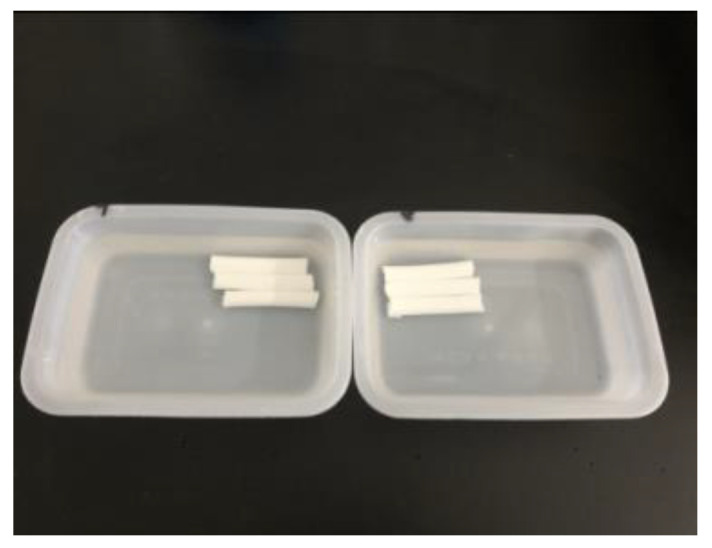
Specimen for water-swelling ratio test.

The prepared slurry was poured into a plastic tube with an inner diameter of 10 mm, and when the slurry formed a gel in the plastic tube and solidified, the curing products were removed at 1 day and cut into specimens with a length of 50 mm, with three specimens in each group. Each specimen was soaked in distilled water (Figure 18). After the swelling volume was stabilized, the water-swelling ratio was calculated as follows:

$$\Delta V=\frac{\left(V_{1}-V\right)-\left(V_{0}-V\right)}{V_{0}-V}\times 100\%$$

where Δ*V* is the water-swelling ratio; *V* is the volume of water in the measuring cylinder in mL; *V*_0_ is the volume of gel and water before the test in mL; and *V*_1_ is the total volume of gel and water after the test in mL.

### 4.6. Grouting Impermeability Repair Test Method

**Figure 19:**
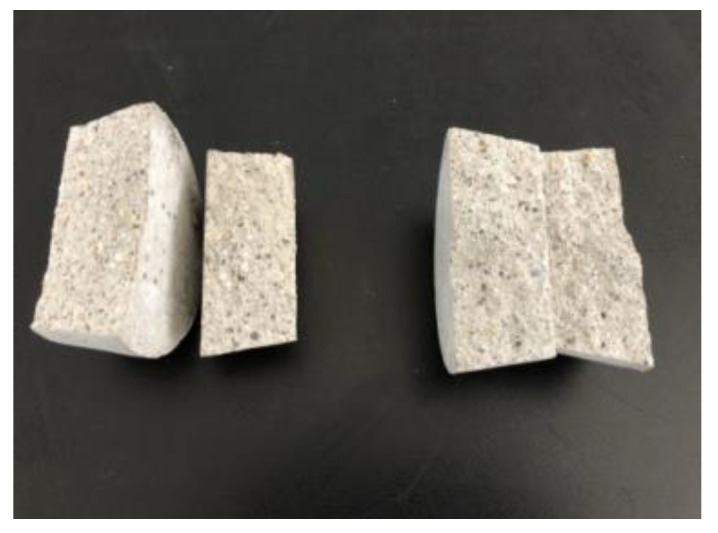
Fractured impermeability test specimens.

**Figure 20:**
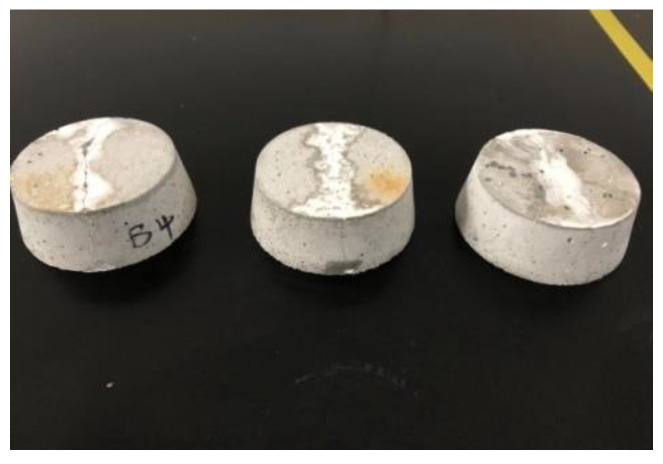
Specimen after grouting.

**Figure 21:**
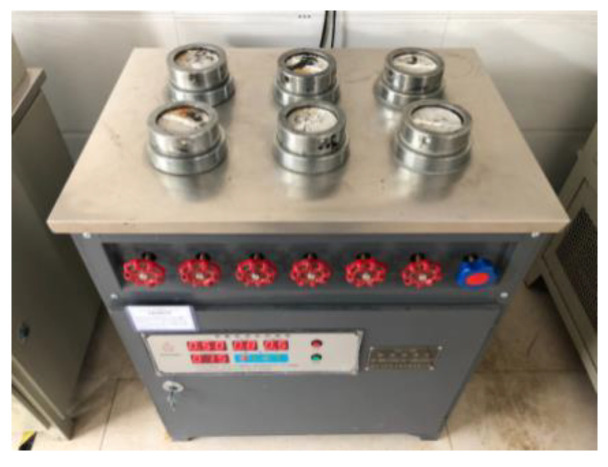
Impermeability tester.

For the impermeability repair test, first, a batch of cement mortar impermeability specimens were fabricated in accordance with the standard of polymer waterproof mortar, and a group of specimens were randomly selected to perform the impermeability test and determine their impermeability grade. After curing under standard conditions for 14 days, the specimens were dried for later use (Figure 19). The prepared double-component acrylate grouting materials were evenly mixed and poured into each specimen along cracks (Figure 20) until the slurry was slightly higher than the specimen surface. After curing under standard conditions for 1 day, the impermeability repair test was carried out at room temperature. The repaired specimen was sealed using sealing materials and placed into an impermeability test mold. The air in the impermeability tester was rejected, and the specimen was placed into the tester (Figure 21). The instrument was started. Pressurizing was performed from 0.2 MPa, and then 0.1 MPa was added every other 1 h until water seepage of the specimen. The water pressure upon the water seepage of the fourth specimen among the six ones was recorded, and the water pressure in case of water seepage (hereafter referred to as seepage pressure) was taken as test data.

### 4.7. Gelation Time Test Method

Time was recorded with a stopwatch, which was pressed to start timing when all components of the acrylate grouting material were mixed in accordance with the mix proportion. Meanwhile, such components were stirred evenly using a glass stirring rod. The stopwatch was pressed again upon slurry reaction and loss of liquidity, and the reading was taken as the gelation time of the acrylate grouting material.

## Figures and Tables

**Table 1:** Orthogonal test results.

Formula No.	Permeability Coefficient (10^−8^ cm/s)	Consolidated Sand Strength (kPa)	Water-Swelling Ratio (%)
1	3.903	215	342
2	3.605	270	315
3	2.960	275	290
4	2.861	295	230
5	3.590	200	335
6	2.684	325	306
7	3.012	260	282
8	2.119	330	265
9	3.791	185	330
10	3.294	245	305
11	2.429	395	286
12	1.805	410	274
13	4.654	175	337
14	3.391	255	328
15	2.007	385	310
16	2.352	415	256

**Table 2:** Range analysis of permeability coefficient.

K_1_	13.33	15.94	11.37
K_2_	11.41	12.97	11.01
K_3_	11.32	8.40	12.26
K_4_	12.40	9.14	13.82
$\bar{K_{1}}$	3.33	3.98	2.84
$\bar{K_{2}}$	2.85	3.24	2.75
$\bar{K_{3}}$	2.83	2.10	3.07
$\bar{K_{4}}$	3.10	2.28	3.46
Range R	2.01	7.54	2.81
Order	B > C > A		

**Table 3:** Range analysis of consolidated sand strength.

K_1_	1055	775	1350
K_2_	1115	1095	1265
K_3_	1235	1315	1045
K_4_	1230	1450	975
$\bar{K_{1}}$	264	194	338
$\bar{K_{2}}$	279	274	316
$\bar{K_{3}}$	309	329	261
$\bar{K_{4}}$	308	363	244
Range R	180	675	375
Order	B > C > A		

**Table 4:** Range analysis of water-swelling ratio.

K_1_	1177	1344	1190
K_2_	1188	1254	1234
K_3_	1195	1168	1213
K_4_	1231	1025	1154
$\bar{K_{1}}$	294	336	298
$\bar{K_{2}}$	297	314	309
$\bar{K_{3}}$	299	292	303
$\bar{K_{4}}$	308	256	289
Range R	54	319	80
Order	B > C > A		

**Table 5:** Influence of accelerator on the water-swelling ratio.

Accelerator	1%	2%	3%	4%	5%	6%
**Water-swelling ratio**	310%	320%	335%	343%	352%	356%

**Table 6:** Influence of initiator on the water-swelling ratio.

Accelerator	1%	2%	3%	4%	5%	6%
**Water-swelling ratio**	302%	310%	340%	347%	Slight damage	Damage

**Table 7:** Orthogonal test scheme.

Formula No.	A Styrene–Acrylic Emulsion (%)	B Crosslinking Agent (%)	C Acrylate (%)
1	0.35	0.15	0.15
2	0.35	0.20	0.20
3	0.35	0.25	0.25
4	0.35	0.30	0.30
5	0.40	0.15	0.20
6	0.40	0.20	0.15
7	0.40	0.25	0.30
8	0.40	0.30	0.25
9	0.45	0.15	0.25
10	0.45	0.20	0.30
11	0.45	0.25	0.15
12	0.45	0.30	0.20
13	0.50	0.15	0.30
14	0.50	0.20	0.25
15	0.50	0.25	0.20
16	0.50	0.30	0.15

## Data Availability

The data sets analyzed or generated during the study have not. yet been archived.
